# Brief ex vivo Fas-ligand incubation attenuates GvHD without compromising stem cell graft performance

**DOI:** 10.1038/s41409-020-0941-2

**Published:** 2020-05-20

**Authors:** Hilit Levy-Barazany, Liat Shachnai-Pinkas, Galina Rodionov, Alex Saar, Michal Rosenzwaig, Liron Gez, Anastasia Rodin, Nitzan Marelly, Michal Abraham, Inbal Mishalian, Hila Wildbaum, Tamar Katz, Yuval Baar, Shai Yarkoni, Ronit Bakimer-Kleiner, Amnon Peled, Tsila Zuckerman, Jerry Stein

**Affiliations:** 1 Cellect Biotherapeutics Ltd., Kfar-Saba, Israel; 2 Goldyne Savad Institute of Gene Therapy, Hebrew University of Jerusalem, Jerusalem, Israel; 3 Hematology Department and Bone Marrow Transplantation Unit, Rambam Health Care Campus, Haifa, Israel; 4 BMT Unit, Department of Pediatric Hematology Oncology, Schneider Children’s Medical Center, Petach Tikva, Israel; 5 Sackler Faculty of Medicine, Tel Aviv University, Tel Aviv, Israel

**Keywords:** Bone marrow transplantation, Cell death and immune response, Haematological diseases, Haematopoietic stem cells

## Abstract

Graft versus host disease (GvHD) remains a limiting factor for successful hematopoietic stem cell transplantation (HSCT). T cells and antigen-presenting cells (APCs) are major components of the hematopoietic G-CSF mobilized peripheral blood cell (MPBC) graft. Here we show that a short incubation (2 h) of MPBCs with hexameric Fas ligand (FasL) selectively induces apoptosis of specific donor T cell subsets and APCs but not of CD34^+^ cells. FasL treatment preferentially induces apoptosis in mature T cell subsets which express high levels of Fas (CD95), such as T stem cell memory, T central memory, and T effector memory cells, as well as T_H_1 and T_H_17 cells. Anti-CD3/CD28 stimulated T cells derived from FasL-treated-MPBCs express lower levels of CD25 and secrete lower levels of IFN-γ as compared to control cells not treated with FasL. FasL treatment also induces apoptosis of transitional, naïve, memory and plasmablastoid B cells leading to a reduction in their numbers in the graft and following engraftment in transplanted mice. Most importantly, ex vivo treatment of MPBCs with FasL prior to transplant in conditioned NOD-scid IL2Rγ^null^ (NSG) mice prevented GvHD while preserving graft versus leukemia (GvL) effects, and leading to robust stem cell engraftment.

## Introduction

Allogeneic hematopoietic stem-cell transplantation (HSCT) can cure a wide range of malignant and non-malignant hematological diseases. Following transplantation of G-CSF-mobilized peripheral blood cells (MPBCs) [1], T cells that are contained in the graft promote hematopoietic engraftment, T-cell immunity and potent graft versus leukemia/lymphoma effects (GvL) [2–4]. A double-edged sword, these T cell can also mediate graft-versus-host disease (GvHD) when they recognize and respond to host allo-antigens presented by host or donor antigen-presenting cells (APCs) [5, 6].

A variety of in vivo or ex vivo modalities are available to prevent GvHD, but these same agents and procedures may attenuate immune reconstitution, increase the risk of infections and/or abrogate T-cell-mediated GvL effects [7, 8].

It has been shown in both preclinical and clinical models that specific T cell subsets exert positive and negative control over the GvHD reaction. In mice, infusion of naïve T cells (CD45RA^+^) induces severe acute GvHD (aGvHD). By contrast, infusion of allogeneic T central memory (CM) induces milder GvHD, and T effector memory cell infusion (EM) does not cause significant GvHD [9–15]. Surprisingly, however, in a single-arm clinical trial in which CD45RA^+^ cells were depleted from allografts, the incidence of aGvHD was not reduced [16, 17].

Fas and its cognate ligand are important regulators of diverse cellular responses. Binding of Fas ligand (FasL; CD178) to Fas (receptor; CD95) initiates a cascade of events culminating in apoptosis [18]. Under physiological conditions, this process curbs exuberant immune responses [19–21]. In the transplant setting, soluble FasL efficiently induces apoptosis in donor anti-host T cells that have been primed by co-culture with irradiated host cells (the mixed lymphocyte reactions (MLR)). Infusion of MLR-primed lymphocytes followed by ex vivo FasL treatment prevents GvHD in preclinical models while preserving antitumor reactivity [22]. Similar results were obtained by ex vivo FasL/MLR depletion of allo-reacting human donor anti-host T cells [23]. Askenasy et al. demonstrated that ex vivo incubation of recipient- naïve donor mouse lymphocytes with FasL causes apoptosis of activated CD8 and CD4 cells with a concurrent increase in the regulatory T-cell (Treg) population [24]. Importantly, these authors showed that treatment of murine recipient-naïve haploidentical splenocytes with FasL in the context of F1 to hybrid transplantation led to lower clinical and histological GvHD scores of skin and gastrointestinal tract as compared with the fatal GvHD seen following the infusion of control splenocytes. Surprisingly, incubation of allografts with FasL (a protein usually associated with apoptosis and cell death) actually facilitated hematopoietic progenitor engraftment and showed trophic effects on primitive hematopoietic precursors. Mice so engrafted demonstrated preserved and potent graft-versus-tumor reactions [24].

As a prelude to the clinical use of FasL to prevent GvHD in patients undergoing stem cell transplantation, we have explored the effects of a brief (2 h) incubation of MPBCs derived from healthy, adult donors with FasL on graft composition (T cell subsets, antigen-presenting cells, in vitro hematopoietic colony formation and CD34^+^ hematopoietic progenitor cells). FasL-treated MPBC’s were infused in immune-deficient mice (both tumor- and non-tumor-bearing) to assess the impact of FasL exposure on the development of both GvHD and GvL. We demonstrate that ex vivo treatment of MPBCs with FasL prior to infusion in NSG mice prevented GvHD in this xenograft setting while preserving both robust stem cell engraftment and GvL.

## Materials and methods

### Apheresis sample collection and FasL treatment

G-CSF mobilized peripheral blood cells (MPBCs) were collected by apheresis from healthy donors at the Schneider Children Medical Center, (Institutional Review Board [IRB] approval No. 0613-14-RMC) or at Rambam Medical Center, (IRB approval No. 506-14-RMB).

Ex vivo incubation of MPBCs with Fas ligand, was performed in a closed infusion bag. In brief, MPBC’s were washed and re-suspended in incubation medium (SCGM, CellGenix, Portsmouth, NH, USA) to which 100 ng/ml of hexameric FasL (MegaFasL, AG-40B-0130-C010, Adipogen, San Diego, CA, USA) was added. After 2 h at 37 °C in 5% CO_2_, cells were washed and re-suspended in transplantation buffer composed of PlasmaLyte A (FKE0324, Baxter Healthcare, Norfolk, UK) and 5% Human Albumin Solution (Zenalb20, Bio Products Laboratory Limited, Hertfordshire, UK). Untreated control MPBCs were stored at 2–8 °C until use.

### Colony-forming unit assay

In vitro colony-forming assays were used as surrogate measures of graft potency. Mononuclear fraction was separated over lymphocyte separation medium (LSM, MP Biomedicals, Santa Ana, California, US). Cells were diluted with IMDM + 2% FBS and MethoCult™ H4034 medium, seeded in a 6 well SmartDish plate (all reagents purchased from StemCell Technologies, Vancouver, BC, Canada) and incubated at 37 °C, in 5% CO_2_, with ≥ 95% humidity for 12–16 days. The number of colonies was expressed per 1 × 10^4^ seeded total nucleated cells (TNCs).

For detection of human colony-forming cells from mouse bone marrow (BM), single-cell suspensions of mouse BM were lysed using ACK Lysis buffer (A10492-01, Thermo Fisher Scientific, Waltham MA, USA) and diluted with IMDM + 2% FBS and MethoCult™ H4534 medium (StemCell technologies, Vancouver, BC, Canada).

### Flow cytometry

Red blood cells from MPBCs or mouse spleen, blood or BM cell suspensions were lysed using ACK lysis buffer (A10492-01, Thermo Fisher Scientific, Waltham MA, USA) and 1 × 10^6^ cells were stained with various combinations of fluorescence- conjugated anti-human antibodies (accept for CD45 that was of either mouse or human origin as indicated in the text). For cell surface staining, cells were incubated with antibodies for 15 min at 4 °C in the dark. Intracellular staining was performed using either Inside Stain Kit or FoxP3 Staining Buffer Set (130-090-477 or 130-093-142, respectively, Miltenyi Biotech, Bergisch Gladbach, Germany) according to manufacturer’s instructions.

The following antibodies were used: anti- mouse CD45 (130-102-412), human CD45 (130-098-143, 130-098-151, 130-098-141, 130-098-148, 130-104-566, 130-110-637), CD34 (130-081-002, 130-090-954), CD90 (130-099-289), CD3 (130-109-460, 130-109-466), CD4 (130-110-680), CD8 (130-109-454), CCR7 (130-108-309), CD45RA (130-108-784, 130-110-637), LFA1 (130-105-437), CD95 (130-104-232), CXCR3 (130-101-378), CCR6 (130-100-377), CD25 (130-109-021), CD33 (130-111-021), CD19 (130-113-642, 130-110-249), CD27 (130-099-499), CD38 (130-108-862), HLA-DR (130-104-825), IL17 (130-100-077), IFN-γ (130-097-944) and FoxP3 (130-098-119) all purchased from Miltenyi Biotech (Bergisch Gladbach, Germany); matched isotype controls were used as negative control.

Induction of early apoptosis was evaluated using Annexin V and 7-Aminoactinomycin D (7-AAD) (BMS500FI and 00-6993 respectively, Invitrogen, Thermo Fisher Scientific, Waltham MA, USA) staining. Early apoptosis was calculated as % AnnexinV^+^/7-AAD^−^ cells of indicated populations.

Data was acquired using MACSQuant analyzer 10 and analyzed using MACSQuant software 2.11 (Miltenyi Biotech, Bergisch Gladbach, Germany). Analysis of CD34^+^ cells was conducted according to the ISHAGE guidelines [25, 26].

### Purification of CD34^+^ or CD3^+^ T cells and T cell activation

CD34^+^ cells were selected from either FasL treated or control MPBCs using CD34 cell-separation microbeads (130-046-703, Miltenyi Biotech, Bergisch Gladbach, Germany). T cell isolation was performed by immunomagnetic negative selection (EasySep, StemCell technologies, Vancouver, BC, Canada). T cells isolated from MPBCs and FasL-treated-MPBCs were stimulated using Human T-Activator CD3/CD28 (111.31D, Dynabeads, Invitrogen, Carlsbad, CA, USA) at a 1:10 bead:cells-ratio. IFN-γ secretion was detected by ELISA assay (Quantikine, R&D systems, Minneapolis, MN, USA) and cells were subjected to flow cytometry assay.

### Xenogeneic GvHD and engraftment models

NOD-scid IL2Rgamma-null (NSG) mice (Jackson Laboratory, Bar Harbor MN, USA) were housed in a pathogen-free facility and handled in accordance with the guidelines of the Animal Care and Use Committee of the Rabin Medical Center, Petach Tikva, Israel. For each experiment 7–10 (acceptable group size based on statistical and ethical considerations) female NSG mice (7–9 weeks) were irradiated with 2 or 2.75 Gy (CLINAC-DBX linear accelerators, Varian Medical System, Palo Alto, CA, USA) 24–48 h prior to infusion of ⁓5 × 10^6^ unfractionated MPBCs (total nucleated cell (TNC)) or 0.1 × 10^6^ purified CD34^+^ cells/mouse, respectively. Mice were randomized according to age and body weight. Monitoring of GvHD clinical score was carried out twice weekly according to the murine clinical grading system described by Cooke et al. [27]. Weight loss, hunched posture, skin lesions, dull fur, and mobility were each assigned scores of 0 (absent), 1 (moderate) or 2 (severe). The investigator was blinded to the group allocation during each experiment.

Mice were sacrificed in case of weight loss ≥20% of initial weight or upon reaching a clinical GvHD score of ≥7. At each study termination endpoint; spleen, blood and BM were harvested, and human cell engraftment was analyzed by flow cytometry. Mice that exhibited <1% hCD45 in their bone marrow or spleen and <0.1% hCD45 in their blood were considered as not engrafted and were excluded from mean calculation.

During the respective scheduled study termination, spleen weight was recorded. The liver of each animal was collected and fixed for at least 48 h in 10% natural buffered formalin (approximately 4% paraformaldehyde) and H&E staining of Paraffin sections was performed. Histopathological changes were scored using semi-quantitative grading (0–4): 0 = No Lesion, 1 = Minimal Change, 2 = Mild Change, 3 = Moderate Change, 4 = Marked Change.

### In vivo GvHD versus leukemia models

Mice were housed in a pathogen-free facility and handled in accordance with the guidelines of the Animal Care and Use Committee of the Hadassah Medical Center, Jerusalem, Israel. Mice were irradiated with 2 Gy, and MV4-11 (10 × 10^6^ cells/mouse, ATCC CRL-9591, Manassas, VA, USA were infused intravenously (IV) within 24 h. After 4–6 h, mice were infused IV with either FasL-treated-MPBCs, control MPBCs or vehicle. Monitoring of GvHD clinical score was carried out twice weekly until 3 weeks post-transplantation, when the mice were sacrificed; blood, BM and spleens were harvested. The leukemic burden and the degree of human leukocyte engraftment were assessed using flow cytometry (MACSQuant Analyzer 10 and MACSQuantify Software 2.11, Miltenyi Biotech, Bergisch Gladbach, Germany). The percentage and relative number of the leukemic cells (hCD45^+^hCD123^+^), human hematopoietic cells (hCD45^+^hCD123^−^) and murine hematopoietic cells (mCD45^+^hCD45^-^hCD123^−^) were evaluated (all antibodies manufactured by Miltenyi Biotech, Bergisch Gladbach, Germany).

### Migration assay and integrin expression

MPBCs control and FasL-treated-MPBCs (2 × 10^5^ TNCs) were placed in the upper compartments of transwell plates (CA-342, Corning, Corning, NY, USA) and their migration in response to 100 ng/ml human CXCL12/stromal derived factor-1 (SDF1) (350-NS-010, R&D Systems) was tested. Chemokine receptor and integrin expression were assessed using anti-CXCR4, anti-LFA1, and anti-VLA4 antibodies (Miltenyi Biotech, Bergisch Gladbach, Germany).

### Statistical analysis

For every figure, statistical tests are indicated.

ANOVA parametric test or two tailed paired T-test for comparison MPBC vs MPBC + FasL samples, data presented as average of three technical replicates for each of at least three independent experiments each performed using individual MPBC donation.

Mann–Whitney test and Log-rank (Mantel–Cox) test for animal studies, at least seven animals/ group was tested.

Two tailed unpaired Student’s *t* test was applied for technical triplicates of individual representative tests. GraphPad Prism version 8.0 (San Diego, CA USA) was used for statistical analyses and figure generation.

## Results

### Brief incubation of G-CSF MPBCs with Fas ligand results in selective reduction of CD3^+^ T cells while maintaining CD34^+^ viability and functionality

**Fig. 1:**
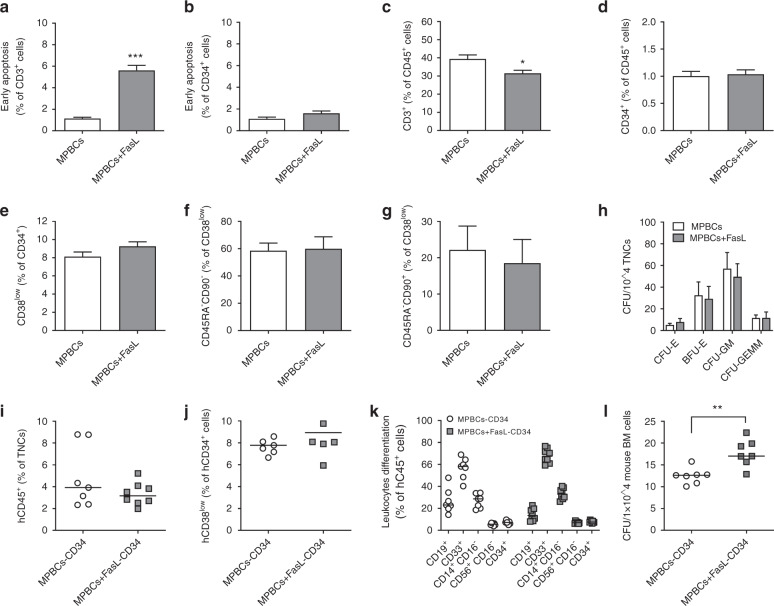
FasL-treatment selectively reduces CD3^+^ cells while CD34^+^ cell number and functionality are maintained. **a**–**h** MPBC graft characterization following FasL treatment. Percentage of annexin V positive **a** CD3^+^ and **b** CD34^+^ cells. **c** Percentage of CD3^+^ and **d** CD34^+^ cells per total CD45^+^ population. HSPCs subpopulations; **e** Immature (CD34^+^CD38^low^), **f** Multipotent progenitors (CD45RA^−^CD90^−^) and **g** self-renewing hematopoietic stem cells (CD45RA^−^CD90^+^). **h** Colony-forming units (CFU) profile of: erythroid progenitor cells (CFU-E and BFU-E), granulocyte-macrophage progenitor cells (CFU-GM) and multipotential granulocyte, erythroid, macrophage, megakaryocyte progenitor cells (CFU-GEMM). Engraftment, differentiation and CFU potential as detected in the BM of γ-irradiated (2.75 Gy) NSG mice, 4 weeks post transplantation of 1 × 10^5^ human CD34^+^ cells: **i** human leukocytes (hCD45^+^) **j** immature hCD34^+^CD38^low^ progenitors and **k** human leukocytes subpopulations: B (hCD19^+^), Myelo-monocytic (hCD33^+^ and CD14^+^CD16^−^), NK (hCD56^+^CD16^−^), HSPCs (CD34^+^) cells and **l** number of human colony-forming cells in the mice BM. Data presented as (**a**–**h**) mean+SD or (**i**–**l**) individual mice and median. (**a**–**d**) *n* = 25 or (**e**–**h**) *n* = 3 individual MPBCs donations and (**i**–**l**) show one representative study out of two, *n* = 7 (MPBC-CD34) and *n* = 8 (MPBC + FasL-CD34) female mice per group transplanted with cells from one individual MPBC donation. Statistical analysis preformed using (**a**–**d**) ANOVA parametric test, (**e**–**h**) paired T-test or (**i**–**l**) Mann–Whitney test. *P < 0.05, **P < 0.01, *** *P* < 0.001.

MPBCs from 25 healthy donors were separately incubated for 2 h with hexameric FasL or with control media. Early apoptosis signal and reduction in the percentage of CD3^+^ T cells were detected in the FasL-treated samples, while CD34^+^ percentage and viability were unaffected (Fig. 1a–d). FasL incubation did not affect the percentage of immature CD34^+^CD38^low^ stem cells, multipotent CD45RA^−^CD90^−^ stem cells, or self-renewing CD45RA^−^CD90^+^ hematopoietic stem cells [28] (Fig. 1e–g). Furthermore, FasL treatment did not reduce the number of erythroid and myeloid colony-forming units that formed in semi-solid, growth factor-supplemented media (Fig. 1h). These results suggest a selective effect of the FasL-treatment on CD3^+^ T cells, with preservation of CD34^+^ progenitor cell viability and clonogenic potential.

Engraftment of HSPCs is dependent on their ability to home to and be retained in marrow niches [29]. The chemokine CXCL12/SDF1 is responsible for the early phases of HSPCs homing and retention in these niches via binding of its cognate receptor, CXCR4. CXCR4-dependent homing and retention of HSPCs also requires integrins such as VLA-4 and LFA-1 [30]. Ex vivo FasL incubation for 2 h did not effect the expression of CXCR4, or of integrins LFA-1 or of VLA-4 on CD34^+^ HSPCs. In functional migration assays, CD34^+^ HSPCs migration towards a CXCL12/SDF1 gradient was not effected by ex vivo FasL treatment (Supplementary Fig. 1a, b).

Engraftment rates of xenotransplanted human leukocytes and hematopoietic progenitor cells (percentage of human CD45^+^ and CD34^+^CD38^low^ cells, respectfully) derived from either control or FasL-treated- MPBC grafts were similar in marrow aspirates from NSG mice 4 weeks after transplantation (Fig. 1i, j). Furthermore, similar percentage of mature human CD19^+^ B cells, CD33^+^ and CD14^+^CD16^−^ myelo-monocytic, as well as CD56^+^CD16^−^ NK cells were observed following control- or FasL-treatment of xenografts (Fig. 1k). A small but statistically significant increase in the number of human CFU was observed in the BM of mice transplanted with CD34^+^ cells derived from FasL-treated-MPBCs compared to the control treatment group at 4 weeks (Fig. 1l). These results demonstrate that ex vivo FasL treatment does not impair short-term engraftment and differentiation of FasL-treated-MPBCs.

### Fas ligand induces apoptosis in specific T cell subsets, reduces expression of activation proteins, and attenuates IFN-γ secretion

**Fig. 2:**
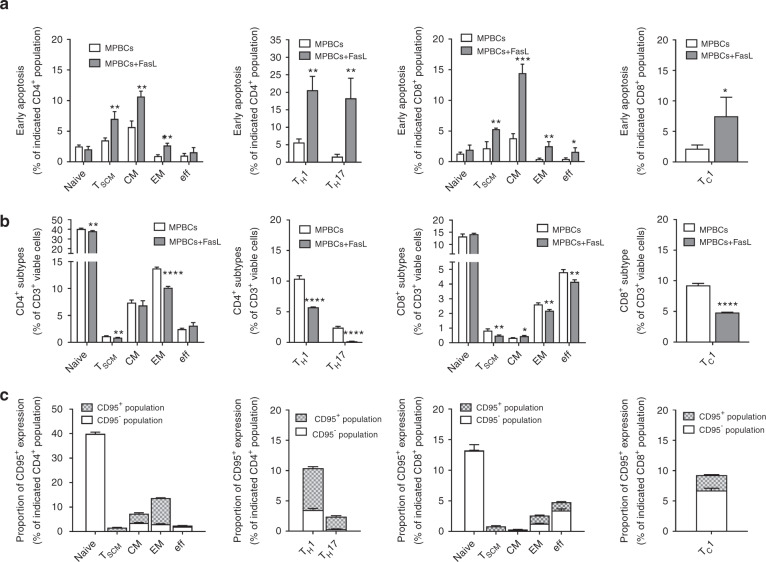
Fas ligand sensitizes T cells to apoptosis. CD4^+^ and CD8^+^ T-lymphocyte subtypes derived from FasL- treated-MPBCs and control MPBCs were analyzed using flow cytometry for (**a**) Percent of early apoptotic cells (Annexin V^+^ stained cells) and (**b**) Percent of viable cells. The following T cell subtypes were analyzed: Naïve (CCR7^+^CD45RA^+^CD95^−^LFA1^low^), T_SCM_ (CCR7^+^CD45RA^+^CD95^+^LFA1^high^), central memory (CM, CCR7^+^CD45RA^−^), effector memory (EM, CCR7^−^CD45RA^−^), effector (eff, CCR7^−^CD45RA^+^) as well as T_H_1 (CD4^+^CXCR3^+^), T_H_17 (CD4^+^CCR6^+^CXCR3^−^) and T_C_1 (CD8^+^CXCR3^+^). **c** Fas (CD95^+^) expressing T-cell populations were analyzed in control MPBC. Results are presented as Mean+SD of experiments performed in triplicate. Due to high donor-dependent variability, one representative experiment out of 3 is presented. Statistical analysis was performed using unpaired, parametric Student’s *t* test *P ≤ 0.05, **P ≤ 0.01, ***P ≤ 0.001, ****P ≤ 0.001.

GvHD is initiated by alloreactivity generated within specific donor-derived T cell subsets; we examined the differential apoptosis-inducing effects of brief ex vivo FasL incubation on T cell subsets present in MPBC grafts. Rates of FasL-induced, early apoptosis were highest among naïve T stem cell memory (T_SCM_), mature helper (T_H_, CD4^+^) and cytotoxic (T_C_, CD8^+^) T cell populations (Fig. 2a, b). Differential FasL sensitivity of each T cell sub-population correlated with the intensity of expression of Fas receptor (CD95) on the cells of each specific population (Fig. 2c).

**Fig. 3:**
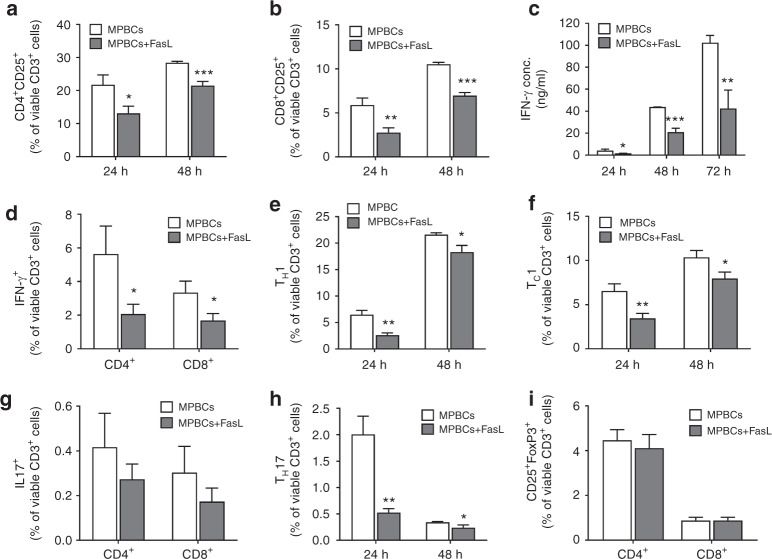
Reduced activation and differentiation of FasL-treated-MPBCs derived T lymphocytes in response to in vitro stimulation. T-lymphocytes isolated from FasL-treated and control MPBCs were stimulated using anti-CD3/CD28 activation beads. **a** Activated T helper (CD4^+^CD25^+^) and **b** T cytotoxic (CD8^+^CD25^+^) cells were quantified 24 and 48 h post stimulation using flow cytometry. **c** Concentration of IFN-γ secreted to the medium of the cultured T-cells was measured 24, 48 and 72 h post stimulation using ELISA. **d** The percentages of CD4^+^ and CD8^+^ IFN-γ secreting (IFN-γ^+^) cells were quantified by flow cytometry 48 h post stimulation. **e** Percent of TH1 (CD4^+^CXCR3^+^) and **f** TC1 (CD8^+^CXCR3^+^) cells were quantified 24 and 48 h post stimulation using flow cytometry. **g** The percentages of CD4^+^ and CD8^+^ IL17 secreting (IL17^+^) cells were quantified by flow cytometry 48 h after stimulation. **h** TH17 (CD4^+^CCR6^+^) T cells populations were quantified 24 and 48 h post stimulation using flow cytometry. **i** Regulatory CD4^+^ and CD8^+^ T cells (CD25^+^FoxP3^+^) were quantified 72 h post stimulation using flow cytometry. Representative results of one independent study out of two is presented. Data presented as Mean+SD, *n* = 3. Statistical analysis was performed using unpaired, parametric t-test; *P ≤ 0.05, **P ≤ 0.01, ***P ≤ 0.001.

To simulate the allo-activation that occurs following infusion of a stem cell graft, CD3^+^ cells derived from MPBC or FasL-treated MPBC were exposed to human T-activator CD3/CD28 beads for 24–48 h; the expression of T-cell activation markers was tested. Incubation of MPBCs with FasL before T cell stimulation reduced the number of CD25^+^ cells (Fig. 3a, b) and significantly reduced both secreted IFN-γ as well as the percentage of cells expressing IFN-γ (Fig. 3c, d). Brief incubation with FasL reduced the percentages of T_H_1, CD4^+^CXCR3^+^ cells, T_C_1, CD8^+^CXCR3^+^ cells and T_H_17, CD4^+^CCR6^+ ^cells (Fig. 3e–h). Interestingly, we found no difference in the percentage of either CD4^+^CD25^+^FoxP3^+^ or CD8^+^CD25^+^FoxP3^+^ T regulatory cell populations following ex vivo treatment with FasL (Fig. 3i).

### FasL-treated-MPBCs attenuate acute GvHD and improve survival following xenogeneic transplantation

**Fig. 4:**
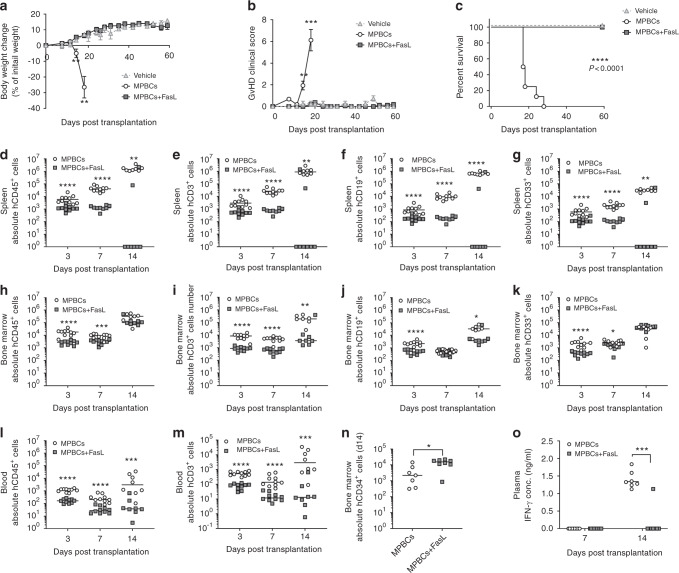
FasL treatment attenuates acute GvHD in a xenogeneic GvHD model. Control MPBCs or FasL-treated-MPBCs (5 × 10^6^/mouse) were transplanted into sub-lethally irradiated (2 Gy) NSG mice (*n* = 10 females/group for day 3 and 7, *n* = 8 females/group for day 14); as control, vehicle (transplantation buffer) was infused (*n* = 2 females/group). The mice were monitored twice a week for (**a**) percentage of body weight change, (**b**) GvHD score and (**c**) survival. Xenograft composition in NSG mice following transplantation of FasL-treated-MPBCs was evaluated 3-, 7- and 14-days post transplantation in the (**d**–**g**) spleen, (**h**–**k**) BM and (**l**–**m**) blood*.* At each indicated termination time point the absolute cell numbers of the following subtypes were measured: **d**, **h**, **l** hCD45^+^, **e**, **i**, **m** hCD3^+^, **f**, **j** hCD19^+^ and **g**, **k** hCD33^+^ (absolute cell number is the product of the percentage of each cell population and the number of cells counted by the flow cytometer after adjusting for the volume of cell suspension). **n** Absolute hCD34^+^ cell number in the BM. **o** Plasma levels of IFN-γ. **a**, **b** Data presented as Mean+SEM, **c** Kaplan Maier survival curve, **d**–**o** Each data point represents an individual mouse, horizontal lines represent the median of each treatment group *n* = 10 for days 3 and 7, *n* = 7 for day 14 female NSG mice per group (results of one representative study out of two are presented). Statistical analysis performed using (**a**–**b** and **d**–**o**) Mann–Whitney test; **c** Log-rank (Mantel–Cox) test. *P ≤ 0.05, **P ≤ 0.01, ***P ≤ 0.001, ****P ≤ 0.0001, *P* ≤ 0.001.

We studied human MPBC engraftment and the development of GvHD in γ-irradiated NSG mice transplanted with either FasL-treated or control grafts. On day 18 post transplantation, the mean clinical GvHD score of control MPBC- infused mice was 6.1 ± 1.0, manifest as weight loss (Fig. 4a), hunched posture, ruffled fur and reduced locomotion. By contrast, the mean clinical GvHD score of mice infused with FasL-treated-MPBCs was zero (*P* < 0.001) (Fig. 4b). GvHD progressed in control-MPBC mice, and none survived to 28 days post-transplantation; by contrast, all mice transplanted with FasL-treated-MPBCs survived at the 60-day benchmark (Fig. 4c) (*P* < 0.0001).

GvHD is triggered early after transplantation when donor T cells home to secondary lymphoid organs, where they undergo activation and expansion before migrating into GvHD target organs [6]. We monitored splenic infiltration by human lymphocytes as a surrogate measure of in vivo T cell expansion and lymph node homing after xenotransplantation of human FasL-treated or control MPBCs in irradiated, immunodeficient mice; human hematopoietic cell engraftment in marrow and peripheral blood were monitored at the same timepoints. Spleens harvested 7 and 14 days after infusion of FasL-treated-MPBCs were significantly smaller (Supplementary Fig. 2a) and contained fewer hCD45^+^, hCD3^+^, hCD19^+^, and hCD33^+^ cells as compared to spleens of mice infused with control MPBC grafts (Fig. 4d–g, respectively). In contrast, hematopoietic marrow engraftment, as measured by the number of human CD34^+^ cells (Fig. 4n) and myeloid precursors in marrow aspirates (Fig. 4k), was similar at day 14 in both control and FasL-treated-MPBC transplanted mice. Additionally, human T and B cell numbers were reduced in the marrow of recipients of FasL-treated-MPBCs as compared to control mice (Fig. 4i and j) as where hCD45^+^ and hCD3^+^ cells in the peripheral blood (Fig. 4l, m). Reduced clinical GvHD scores of FasL-treated grafts were paralleled by lower IFN-γ plasma levels (Fig. 4b, o) and a reduction in histologic signs of GVHDs in the liver parenchyma of these mice (Supplementary Fig. 2b, c).

### Fas ligand induces apoptosis in B cell subsets

**Fig. 5:**
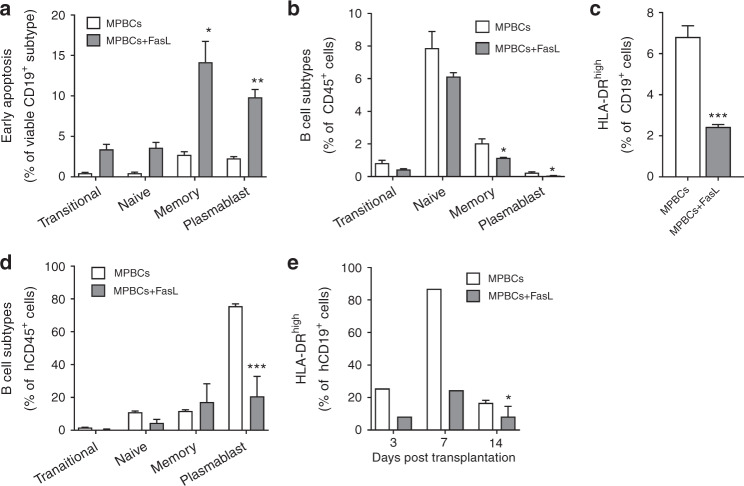
FasL treatment changes the composition of the B cell compartment. In vitro (**a**–**c**) and in vivo (**d**–**e**) studies demonstrate the effect of FasL treatment on B cells. **a** Early apoptosis (AnnexinV^+^/7-AAD^−^) and **b** percentages of B cell subtypes [Transitional (CD27^−^CD38^+^), Naïve (CD27^−^CD38^−^), Memory (CD27^+^CD38^−^), and Plasmablast (CD27^+^CD38^+^)], **c** percentage of HLA-DR^high^ expressing B cells in control- and FasL-treated MPBCs. **d** Percentage of human B-lymphocytes subtypes in spleens 14 days post transplantation of either control or FasL-treated-MPBCs into NSG mice and **e** the percentage of HLA-DR^high^ expressing human-B-lymphocytes 3, 7 and 14 days post transplantation. Data presented as Mean +SEM. Statistical analysis was performed using (**a**–**c**) Student’s *t* test and (**d**, **e**) Mann–Whitney test; *P < 0.05, **P < 0.01, ***P < 0.001, *n* = 10 for days 3 and 7, *n* = 7 for day 14 female NSG mice per group.

Accumulation of T cells in the spleens of control MPBCs recipients suggests the presence of local antigen-presenting cells (APCs). Indeed, we noted a parallel increase in splenic human T and B cells in our xenograft recipients following the administration of control MPBCs, and attenuation of this process in recipients of FasL-treated-MPBCs (Fig. 4f). As B cells serve as surrogate APC’s in xenograft models [31, 32], B cell subsets were evaluated in FasL-treated-MPBC grafts (Fig. 5a–c). FasL treatment lead to increased apoptosis of transitional, naïve, memory and plasmablastoid B cells and a reduction in their relative percentage within the treated grafts (Fig. 5a, b). CD95 expression was noted in the most affected cell populations (data not shown). Ex vivo FasL treatment selectively reduced the percentage of HLA-DR^high^ B cells (Fig. 5c). Two weeks after transplantation in immune-deficient mice, transitional, naïve and plasmablastoid B cells were reduced in recipients of FasL-treated-MPBCs (Fig. 5d) as compared to recipients of control grafts. HLA-DR^high^ B cells were selectively decreased in spleens of mice transplanted with FasL-treated-MPBCs as compared to mice transplanted with control MPBCs (Fig. 5e), mirroring the results of the ex vivo experiments noted above.

### FasL treatment maintains GvL activity while preventing GvHD

**Fig. 6:**
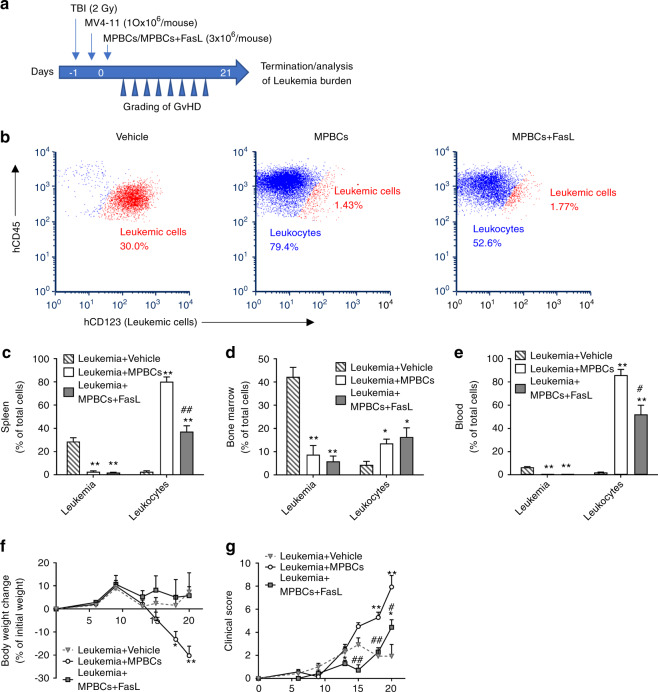
FasL prevents GvHD but preserves GvL in vivo. **a** Graft vs. leukemia model: 1 day following sub-lethal γ-irradiation (2 Gy), 10 × 10^6^ MV4-11 leukemic cells were administered by intravenous (IV) bolus injection followed 4–6 h later by infusion of either 3 × 10^6^ MPBCs or FasL-treated-MPBCs. Animals were evaluated twice weekly for clinical signs of GvHD. Human hematopoietic cell engraftment and leukemic burden were assessed 3 weeks post transplantation in blood, BM and spleen by flow cytometry. **b** Representative flow cytometry dot plot analysis of splenocytes, displaying leukemic cells (hCD45^+^hCD123^+^) and human leukocytes (hCD45^+^hCD123^−^). Data are representative of one of two independent experiments. Percentage of human leukemic cells and human leukocytes in the (**c**) spleen, (**d**) BM and (**e)** blood. (**f)** Body weight change and (**g**) clinical GvHD scores of NSG leukemic mice injected with FasL-treated or control MPBC. Data are presented as Mean + SEM, (*n* = 7 female NSG mice per group). *P < 0.05, **P < 0.01 versus Vehicle treated group and ^#^*P* < 0.05, ^##^*P* < 0.01 versus MPBCs control group (Mann–Whitney test).

The presence of T cells in the transplanted graft promotes both engraftment and GvL [33]. To study the effect of FasL treatment on GvL in vivo, we developed a novel model for concurrently testing GvHD and GvL in NSG mice. MV4-11 human leukemic cells were administered intravenously into γ-irradiated NSG mice on day 0 (10 × 10^6^ cells/mouse), and either FasL-treated or control MPBCs (3 × 10^6^ TNCs/mouse) were infused 4–6 h later. GvHD scores were recorded twice weekly for three weeks and at the timepoint at which the mice were sacrificed; leukemic burden in the marrow, spleen and blood was assessed using antibodies to human CD123 (Fig. 6a, b). As compared to mice infused with sham stem cell grafts (vehicle), leukemic burden was similarly diminished in the spleen, marrow, and blood of mice co-transplanted with either FasL-treated-MPBCs or control MPBCs (*P* < 0.01 either transplant vs. sham) (Fig. 6c–e), suggesting that xenograft-mediated GvL was not compromised by FasL treatment. As in the previous experiments (see Fig. 4), recipients of FasL-treated-MPBCs continued to be robust, with no weight loss and with lower GvHD clinical scores as compared to mice receiving control MPBCs (Fig. 6f, g). Of note, vehicle infused mice showed worsening clinical scores due to increasing leukemic burden, and not due to GvHD (Fig. 6g). In summary, FasL permits uncoupling of GvHD and GvL effects in this xenograft model.

## Discussion

In this study, we have shown that brief incubation of human stem cell grafts with FasL uncouples GvHD and GvL effects without attenuating the engraftment potential of the cells in irradiated, immune-deficient mice. Attenuation of GvHD by brief incubation of otherwise unmanipulated human stem cell grafts with multimerized FasL occurred even in the absence of prior priming of donor T cells with host antigens. FasL incubation increased apoptosis of IFN-γ producing T cells and of cell populations expressing high levels of CD95, including naïve derived T_SCM_, mature helper (T_H_, CD4^+^) and cytotoxic T cells (T_C_, CD8^+^), memory and effector T cells, and T_H_1/T_C_1 and T_H_17 cells (Fig. 2c). Recent studies suggest that acute GvHD is mediated in part by mature donor T_H_1 and T_H_17 cells present in the allograft that recognize minor or major histocompatibility disparities between donor and host [34, 35]. In addition to fully differentiated T-cell populations, T_SCM_ includes a minor population of self-renewing cells which expands in the host early after transplantation [36–38], and the dynamics of T_SCM_ expansion and contraction correlates with GvHD-free survival [39, 40]. These cells are at the cusp of the transition between naïve and effector state and may serve as a cellular reservoir for GvHD-inducing cells. It is not surprising, therefore, that partial depletion of these cells from the graft by FasL treatment would ameliorate GvHD. Tandem depletion of less differentiated T_SCM_ cells and mature, IFN-γ secreting T cells may shape the alloreactive post-transplant T cell milieu, reducing the risk of severe GvHD. Recent descriptions of the role of T cell collectivity and the influence of small T cell populations on antigen response suggest that even partial depletion of small but influential T cell subpopulations within the graft’s lymphocyte compartment may have substantial effects on post-transplantation alloreactivity. Further elucidation of the rules of T cell social behavior will likely continue to inform the future development of novel GvHD therapeutics [41].

During stem cell engraftment, donor T cells interact with both host and donor APCs; host APCs present host major and minor histocompatibility antigens to donor T cells priming the acute GvHD reaction [6]. T-cell recognition of MHC molecules is species-restricted; human TCRs do not recognize mouse MHC. GvHD in xenogeneic transplant of human MPBCs into immunodeficient NSG mice ensues when HLA proteins on APCs contained in the graft process and present mouse antigens to T cells that are also contained within the graft [31, 32]. Indeed, in our model, donor-derived T and B cells co-accumulate in the spleen presaging the appearance of GvHD (Fig. 4d). Brief incubation of MPBCs with FasL resulted in increased apoptosis among transitional, naïve, memory and plasmablastoid B cells, leading to a reduction in their percentage within the treated graft (Fig. 5a, b), and, eventually, within the spleen of transplanted mice (Fig. 5d, e). Furthermore, FasL treatment selectively reduced the percentage of putative antigen-presenting HLA-DR^high^ expressing B cells in vitro and in vivo (Fig. 5c, e). Induction of apoptosis and reduction in the number of myeloid APCs that express HLA-DR^high^ was found in vitro as well as in vivo in the spleen of mice transplanted with FasL-treated grafts (Supplementary Fig. 3). This FasL-induced decrease in antigen-presenting capacity corelated with lower GVHD scores and mortality.

We did not directly measure markers of inflammation in treated and control mice, but speculate that FasL-mediated changes in graft composition may reduce levels of inflammation and promote short-term HSC engraftment and hematopoietic recovery.

Host APCs play an important role in the balance between the induction of GvHD and the development of tolerance [42]. Phagocytosis of apoptotic cells converts immature dendritic cells (DCs) into tolerogenic DCs, abrogating GvHD [43, 44]. Several different techniques can be used to increase the number of apoptotic cells within the graft. Apoptosis can be induced by incubation of the graft with a photoactivated dye (8-Methoxypsoralen) followed by exposure to ultraviolet light, a process which is exploited clinically in extracorporeal photopheresis (ECP) in GvHD patients unresponsive to conventional immunosuppressive drugs [45], and FasL-induced apoptosis of lymphocytes contained the grafts used in these studies may lead to immunomodulation of macrophages and DCs and reduced rates of GvHD [46, 47]. Support for this theory is provided by suppression of GvHD by FasL pretreatment of transplanted grafts in a model that uses syngeneic mice [24].

Ex vivo T cell depletion from donor grafts has been used to prevent GvHD for more than 40 years. Iterations of this technique include positive selection of CD34^+^ hematopoietic stem cell progenitors and immunomagnetic depletion of target T cell populations [7]. Other approaches have been developed to deplete GvHD causing cells in vivo using anti-T-cell antibodies, or the administration of post-transplant cyclophosphamide [48]. Despite the positive effects of GvHD reduction seen after the administration of these modalities, patients often pay a price for T cell depletion, manifest by delayed immune recovery and attenuation of GvL effects. Brief incubation (2 h) of MPBCs with hexameric Fas ligand, offers a novel ex vivo technology to treat GvHD by selectively depleting specific subsets of T cells, together with the ability to attenuate donor and host allo-antigen presentation involved in GvHD. Engraftment in our murine model is robust following transplantation of treated graft, and the graft retains its immune reconstitution and anti-leukemic effects A phase I clinical trial using such graft in adults undergoing stem cell transplant for the treatment of hematological malignancies is currently underway (NCT02828878).

## Supplementary information

Supplementary Figure Legends

Suuplementary Figure 1

Suuplementary Figure 2

Suuplementary Figure 3
