# Supplementary material for: Brief ex vivo Fas-ligand incubation attenuates GvHD without compromising stem cell graft performance
Source: Bone Marrow Transplant. 2020 May 20;55(7):1305–16. doi: 10.1038/s41409-020-0941-2 (PMC7329633; doi:10.1038/s41409-020-0941-2)
Supplement: Supplementary file 1 — Supplementary Figure Legends [file 41409_2020_941_MOESM1_ESM.pdf]

Supplementary Figure Legends

Supplementary Figure 1. FasL treatment compromises neither quality nor quantity of CD34^{+} hematopoietic stem and multipotent progenitor cells. (A) Adhesion molecules and chemokine receptor expressing cell profile; CXC-chemokine receptor 4 (CXCR4), lymphocyte function-associated antigen 1 (LFA1) and very late antigen 4 (VLA4). (B) CD34^{+} cell's migration towards SDF-1 gradient. Data presented as Mean+SD of n=4 (A) and n=5 (B) individual MPBC donations, statistical analysis preformed using paired T-test.

Supplementary Figure 2. FasL treatment prevents GvHD-related spleen and liver injury. (A) spleen weight of NSG mice 3-, 7- and 14-days post transplantation (B) Mean values of hepatocytic necrosis histological score and (C) representative images of histological sections of livers removed from mice 14 days post MPBCs transplantation and stained with hematoxylin and eosin (yellow arrow point to a hepatocytic necrosis lesion). Data presented as Mean+SEM, n=5, statistical analysis performed using Mann Whitney test; $^{**}P \leq 0.01$.

Supplementary Figure 3. FasL effect of Myeloid population ex-vivo as well as in-vivo. (A) Early apoptosis and (B) percentage of HLA-DR^{high} myeloid cells in the pre-transplanted control and FasL-treated grafts (C) HLA-DR^{high} human myeloid cells harvested from the spleen of NSG mice 14 days post transplantation. Data is presented as Mean+SEM. Statistical analysis was performed using (B-D) Student's t-test and (E and F) Mann Whitney test; $^{**}P<0.01$, $^{****}P<0.0001$.
