# Supplementary figures and images for: Brief ex vivo Fas-ligand incubation attenuates GvHD without compromising stem cell graft performance
Source: Bone Marrow Transplant. 2020 May 20;55(7):1305–16. doi: 10.1038/s41409-020-0941-2 (PMC7329633; doi:10.1038/s41409-020-0941-2)

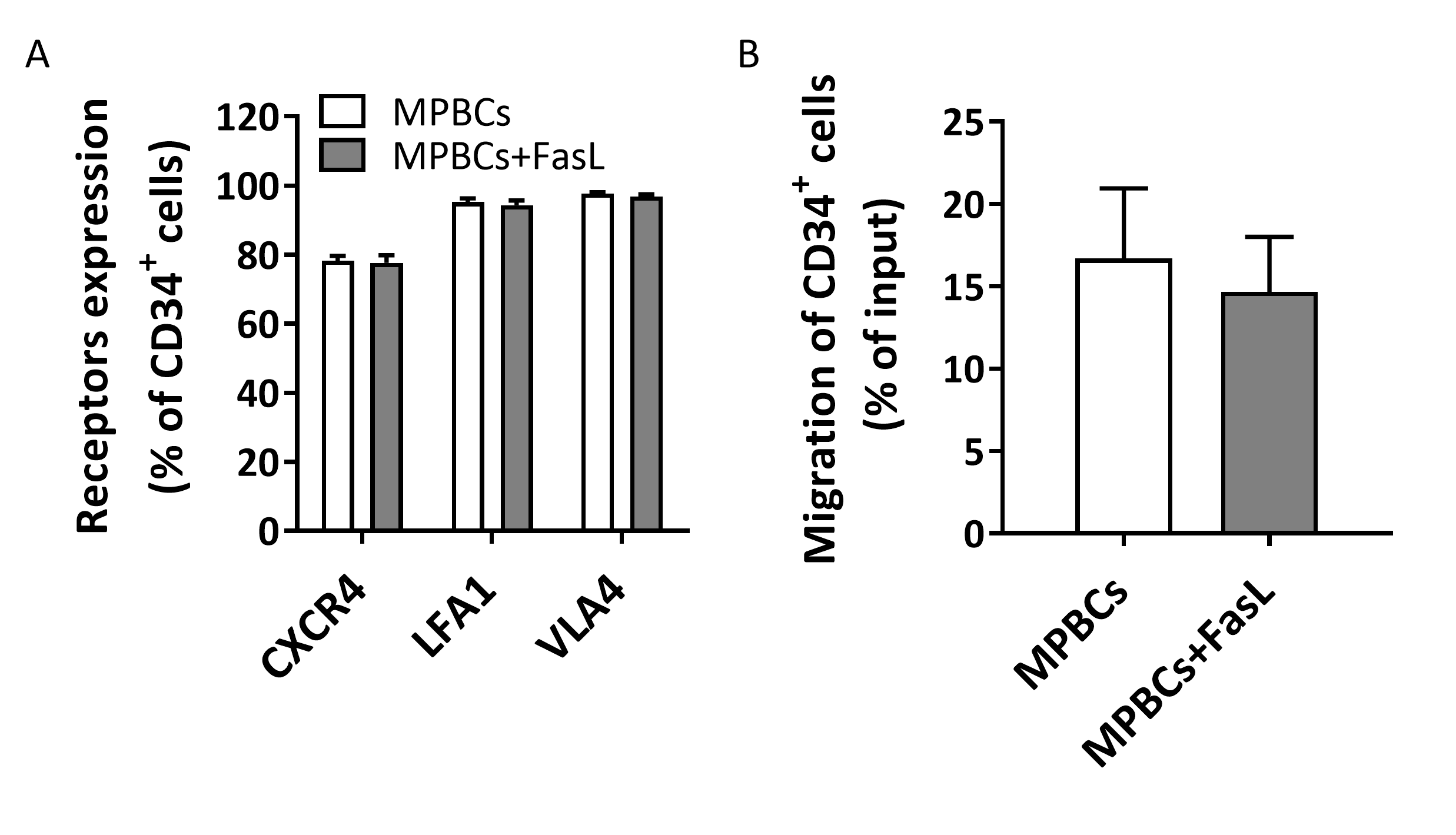

Supplement: Supplementary file 2 — Suuplementary Figure 1 [file 41409_2020_941_MOESM2_ESM.tif]

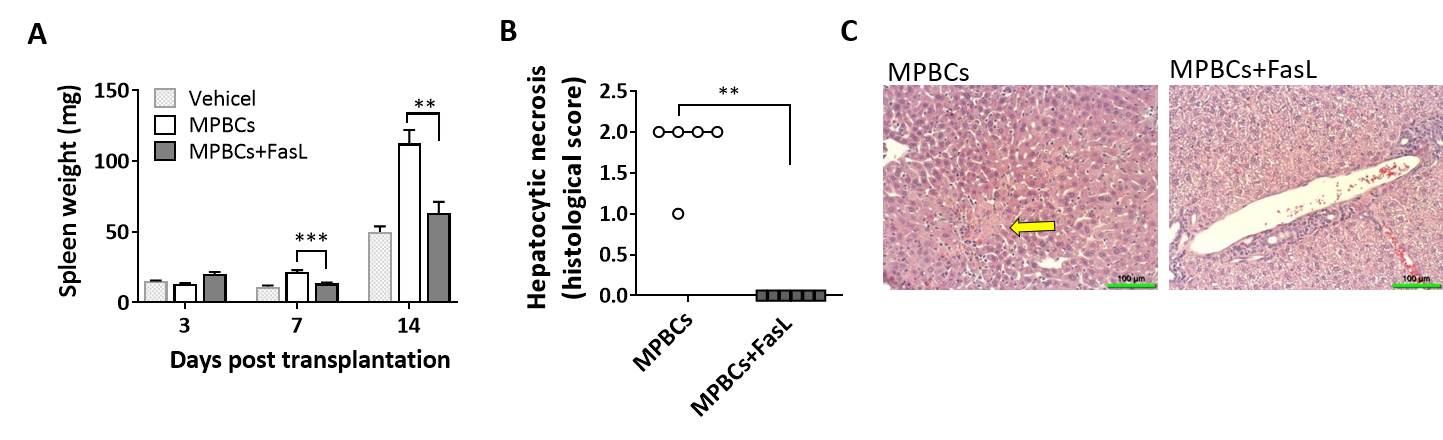

Supplement: Supplementary file 3 — Suuplementary Figure 2 [file 41409_2020_941_MOESM3_ESM.tif]

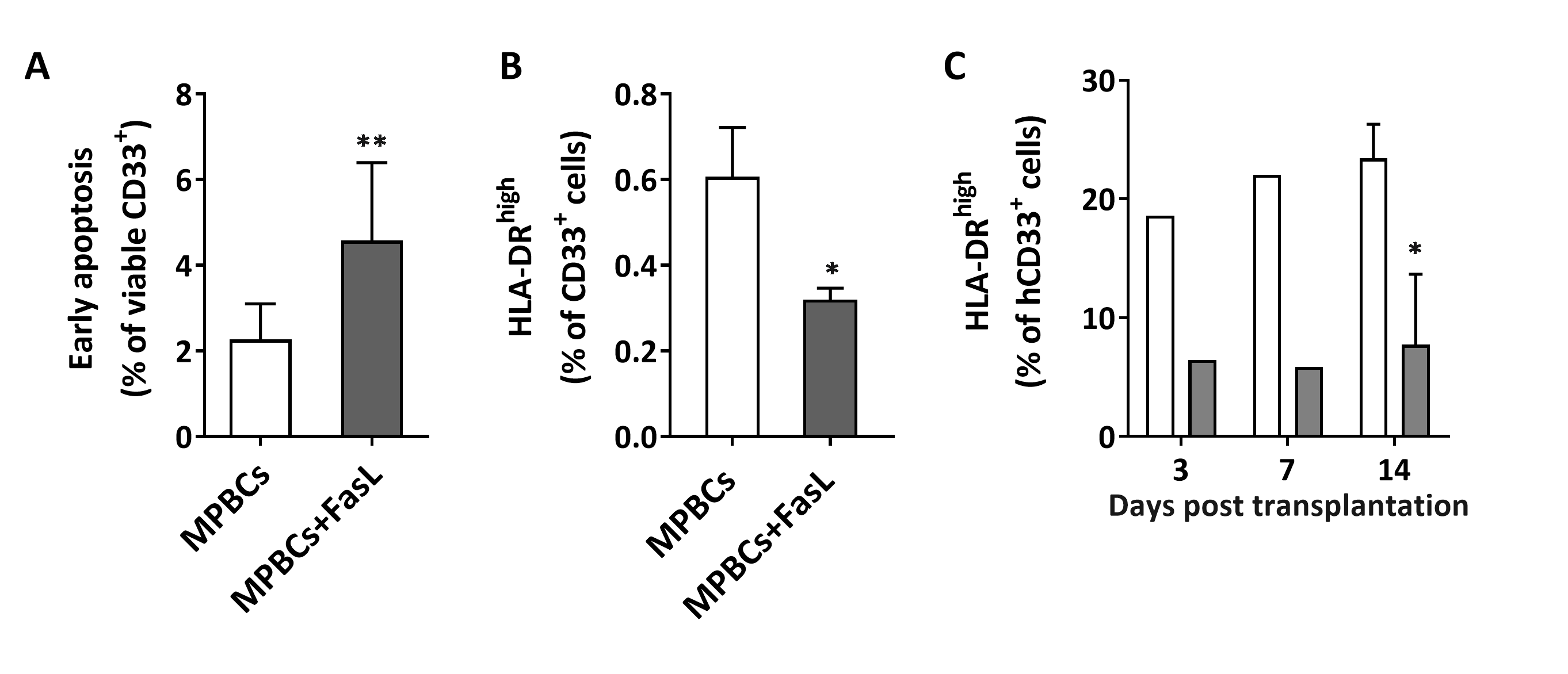

Supplement: Supplementary file 4 — Suuplementary Figure 3 [file 41409_2020_941_MOESM4_ESM.tif]
